# Nitric Oxide Implication in Potato Immunity to *Phytophthora infestans* via Modifications of Histone H3/H4 Methylation Patterns on Defense Genes

**DOI:** 10.3390/ijms23074051

**Published:** 2022-04-06

**Authors:** Andżelika Drozda, Barbara Kurpisz, Magdalena Arasimowicz-Jelonek, Daniel Kuźnicki, Przemysław Jagodzik, Yufeng Guan, Jolanta Floryszak-Wieczorek

**Affiliations:** 1 Department of Plant Physiology, Faculty of Agronomy, Horticulture and Bioengineering, Poznan University of Life Sciences, 60-637 Poznan, Poland; andzelika.drozda@up.poznan.pl (A.D.); barbara.kurpisz90@gmail.com (B.K.); daniel.kuznicki@up.poznan.pl (D.K.); yufeng.guan@amu.edu.pl (Y.G.); 2 Department of Plant Ecophysiology, Faculty of Biology, Adam Mickiewicz University in Poznan, 61-614 Poznan, Poland; arasim@amu.edu.pl (M.A.-J.); przemyslaw.jagodzik@amu.edu.pl (P.J.)

**Keywords:** nitric oxide, stress-responsive gene regulation, histone lysine, arginine methylation, potato hypersensitive response, late blight

## Abstract

Nitric oxide (NO) is an essential redox-signaling molecule operating in many physiological and pathophysiological processes. However, evidence on putative NO engagement in plant immunity by affecting defense gene expressions, including histone modifications, is poorly recognized. Exploring the effect of biphasic NO generation regulated by S-nitrosoglutathione reductase (GNSOR) activity after avr *Phytophthora infestans* inoculation, we showed that the phase of NO decline at 6 h post-inoculation (hpi) was correlated with the rise of defense gene expressions enriched in the TrxG-mediated H3K4me3 active mark in their promoter regions. Here, we report that arginine methyltransferase PRMT5 catalyzing histone H4R3 symmetric dimethylation (H4R3sme2) is necessary to ensure potato resistance to avr *P. infestans*. Both the pathogen and S-nitrosoglutathione (GSNO) altered the methylation status of H4R3sme2 by transient reduction in the repressive mark in the promoter of defense genes, *R3a* and *HSR203J* (a resistance marker), thereby elevating their transcription. In turn, the PRMT5-selective inhibitor repressed *R3a* expression and attenuated the hypersensitive response to the pathogen. In conclusion, we postulate that lowering the NO level (at 6 hpi) might be decisive for facilitating the pathogen-induced upregulation of stress genes via histone lysine methylation and PRMT5 controlling potato immunity to late blight.

## 1. Introduction

The last three decades of intensive research on nitric oxide in plants has highlighted nitric oxide (NO) engagement in different aspects of development and stress-related responses. The wide range of NO bioactivity depends on its diffusion properties, high reactivity affecting the function of a multitude of cellular proteins, as well as its concentration. Nitric oxide has been documented as a critical redox signaling molecule effective in triggering plant responses against a wide range of pathogens during both pathogen-associated molecular pattern (PAMP) triggered immunity (PTI) and a particular effector-triggered immunity (ETI) [1,2]. During the first hours after a pathogen challenge, NO is generated by a plant in response to the pathogen attack as a local NO burst, stimulating a different sequence of defense events [3,4,5,6,7,8]. Nitric oxide generation in synergy with reactive oxygen species (ROS) may lead to the formation of peroxynitrite (ONOO¯) which, at the molecular level, may constitute the signaling mode of NO action via tyrosine residue nitration in proteins [9,10,11,12,13]. S-nitrosation, based on the NO equivalent transfer to cysteine thiol, is the subsequent reversible redox and NO-dependent post-translational protein modification (PTM) regulating the activity of many proteins, including R and PR (pathogenesis-related) proteins working in cooperation with transcription factors [14,15,16,17]. Some of the essential NO activities result from its covalent binding to the ferrous heme in proteins or the formation of nitrosyl–iron complexes (DNICs) [18]. NO in direct or indirect interactions with target proteins and other NO-dependent post-stress processes can affect plant immunity. Sometimes, when a stress stimulus overpowers the physiological response buffer, plant survival can involve epigenetic regulations [19].

Epigenetic control of gene expression relies on DNA methylation, the RNA-directed DNA methylation (RdDM) pathway with small non-coding interfering RNA (siRNA), and histone-modifying complexes, which regulate chromatin structure [20]. Under abiotic and biotic stresses, chromatin is dynamically modulated between the transcriptionally repressed and active states to regulate gene expression. Histone proteins are particularly susceptible to various PTMs, including methylation; however, the role of histone modifications during stress is not as straightforward as that of DNA methylation [21].

Depending on the site and extent of modification (mono-, di-, or tri-methylation) on lysine or arginine residues, histone methylation can contribute to the active or inactive conformation of chromatin. The state of lysine methylation can be achieved by a balance between the action of targeted methyltransferases (HMTs) and demethylases (KDMs), which can remove methyl groups from histone proteins. Most lysine methyltransferases contain a conserved SET domain, based on the three histone lysine methyltransferases, i.e., the suppressor of variegation [SU(VAR)3-9], enhancer of zeste [E(z)], and trithorax [Trx]. The trithorax subfamily proteins involved in transcription activation catalyze the di- or tri-methylation of H3K4, whereas the enhancer of the zeste subfamily, which is the active subunit of the polycomb repressor complex (PRC) harboring curly leaf (CLF), targets the di- and tri-methylation of H3K27 and inhibits gene expression [22,23,24,25].

In turn, protein arginine methylation is catalyzed by a group of highly conserved protein arginine methyltransferases (PRMTs), of which PRMT5 has been well characterized in plants. PRMT5 is also named SKB1 (kinase binding protein 1) or CAU1 (calcium under accumulation 1), because protein arginine methyltransferase 5 is a type II arginine methyltransferase that catalyzes Arg symmetric dimethylation at the arginine residue R on histone H4 (H4R3sme2) [26,27]. PRMT5 methylates a large pool of target substrates, including histone and non-histone proteins, to regulate gene expression, RNA elongation, pre-mRNA splicing, protein regulation, and cell stability [28,29,30,31,32,33,34]. Increasing evidence suggests that PRMT5 regulates developmental processes and plant responses to environmental stresses. Study of *PRMT5* mutants revealed that *AtPRMT5* deficiency causes pleiotropic phenotypes, including growth inhibition, dark green and curled leaves, delayed flowering and reduced sensitivity to vernalization, hypersensitivity to salt, and drought [31,32,35].

Due to its pleiotropic functions, it could be assumed that PRMT5 is subject to multi-level regulation and modification. It has been found that human PRMT5 is phosphorylated at several residues, and this crosstalk between kinases and arginine methyltransferases may play a pivotal role in modulating different cellular functions of PRMT5 [36]. Additionally, experimental evidence in plants has shown that NO positively regulates PRMT5 function under stress conditions via the S-nitrosation of PRMT5 at cysteine 125, promoting methyltransferase activity associated with salt stress tolerance [37].

Compared with the well-established knowledge about the pathway of NO signaling to specific downstream effects in the plant, studies on epigenetic regulation by NO-mediated chromatin-modifying enzymes, altering histone post-translational modification, DNA methylation, and microRNA expression are in their infancy. To date, several reports have been published on the potential NO-dependent effects on chromatin structure affecting gene expression in plants [38,39,40,41,42,43]. The experimental evidence was focused on exploring NO regulation via the tyrosine nitration or S-nitrosation of histone deacetylases (HDACs), of which the downregulation enhances acetylation and makes chromatin more accessible for transcription factors [44,45,46,47]. A study on GSNOR1-mediated histone and DNA methylation has been published recently, revealing the complex picture involving a new NO function as an epigenetic mediator in plants [48].

Our understanding of the molecular mechanisms of epigenetic variation in crop improvement strategies, including disease resistance, is rapidly growing [49,50,51,52]. Most of the recognized epigenetic mechanisms are related to model plants. In the present study, we focused on exploring histone modifications occurring in potato responses to *Phytophthora infestans*, the causative agent of late blight disease. Late blight remains the most devastating disease in potato, and the direct cost of plant protection, together with lost production, is assessed at over USD 5 billion per year globally [53]. Potato *P. infestans* epigenetic modifications should be better recognized and addressed for future potato resistance improvement.

Thus, our research provides new insight into NO-associated potato immunity to avr *P. infestans*, including redox- and time-dependent crosstalk between histone lysine and arginine methylation, contributing to reprogramming defense genes. Our findings revealed the molecular dialog between biphasic NO generation and PRMT5 linked with reversible deposition of the repressive H4R3sme2 mark on the resistance genes promoter, thus regulating their transcription.

## 2. Results

### 2.1. Biphasic NO Production under GSNOR Controlling

**Figure 1:**
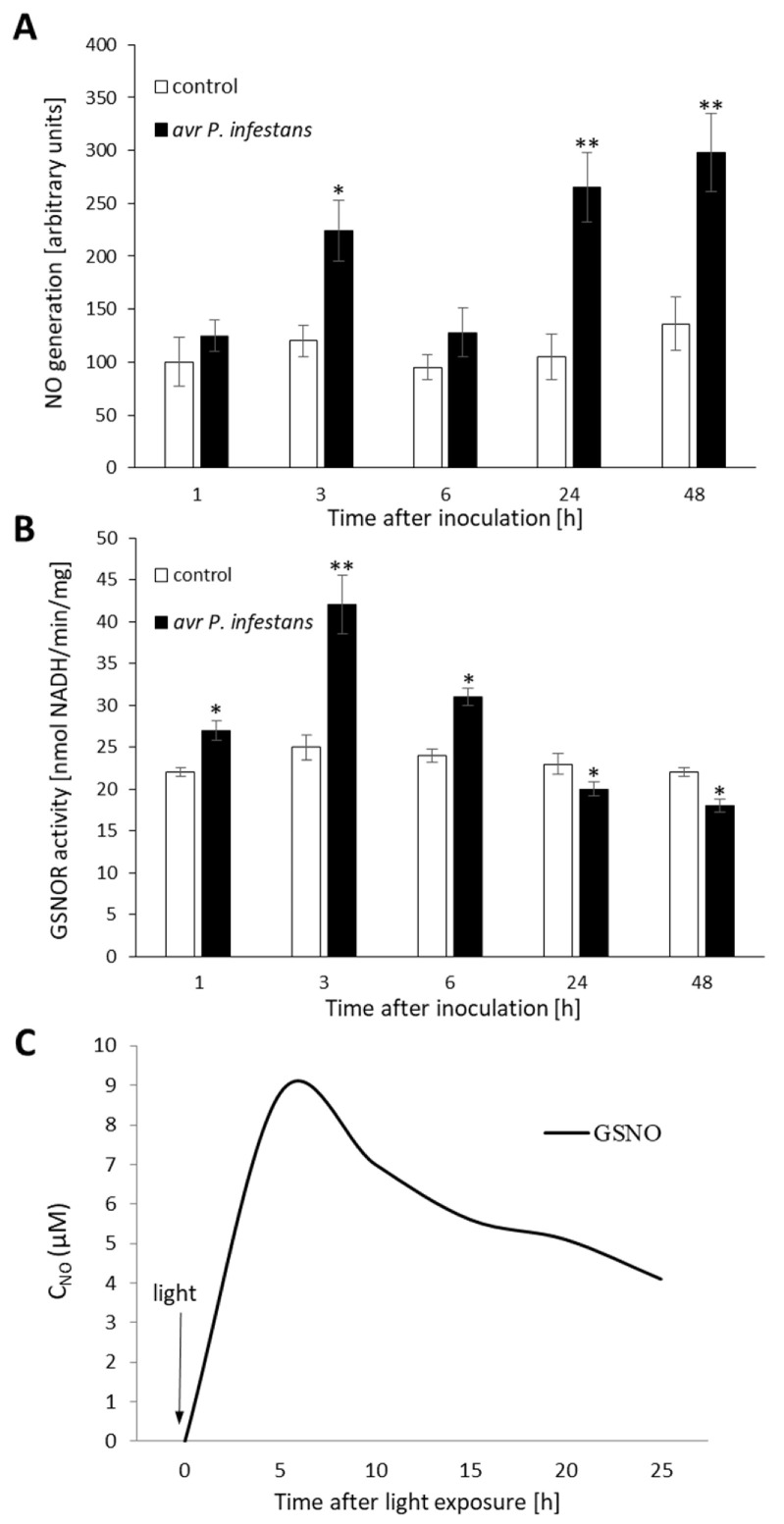
Nitric oxide burst (**A**) and GSNOR activity in potato avr *P. infestans* interaction (**B**), concentration–time traces of NO emission from GSNO under continuous illumination (**C**). Conditions applied in electrochemical NO detection: donor concentration, 250 µM; electrolyte, phosphate buffer pH 7.4; light, polychromatic (white), illumination intensity 180 µmol photons m^−2^ s^−1^; starting temperature, 20 °C; final temperature, 26 °C. Values represent the means of data ± SD of three independent experiments. Asterisks indicate values that differ significantly from control leaves at α < 0.05 (*) and α < 0.01 (**).

To determine the impact of pathogen-induced NO burst on epigenetic variations in potato plants, the level of NO was measured in leaves inoculated with avr *P. infestans*. The obtained data revealed two waves of NO overproduction after pathogen inoculation. The biphasic NO profile consisted of an initial sharp increase (at 3 hpi), subsequent decline (at 6 hpi), and a second (at 24–48 hpi) stronger phase of NO generation (Figure 1A). NO formation cooperated with S-nitrosoglutathione reductase (GSNOR) activity. The primary function of GSNOR is based on the regulation of intracellular resources of GSNO and NO/SNOs. Our data suggest that GSNOR activity increased gradually up to 3 hpi, then decreased (Figure 1B), and corresponded to the early timing of NO formation.

To compare the effect of endogenous NO generation after avr *P. infestans* inoculation with exogenous NO on histone methylation changes, potato leaves were treated with 250 µM GSNO. The enhanced NO emissions from GSNO solution were found between 3 and 24 h after turning on the light, at a half-life t½ = ca. 7 h (Figure 1C). These data are in accordance with our previous study [54]. Moreover, to evaluate NO’s contribution to a given process, leaves were treated independently with cPTIO (the NO scavenger) or GSH (additional control).

**Figure 2:**
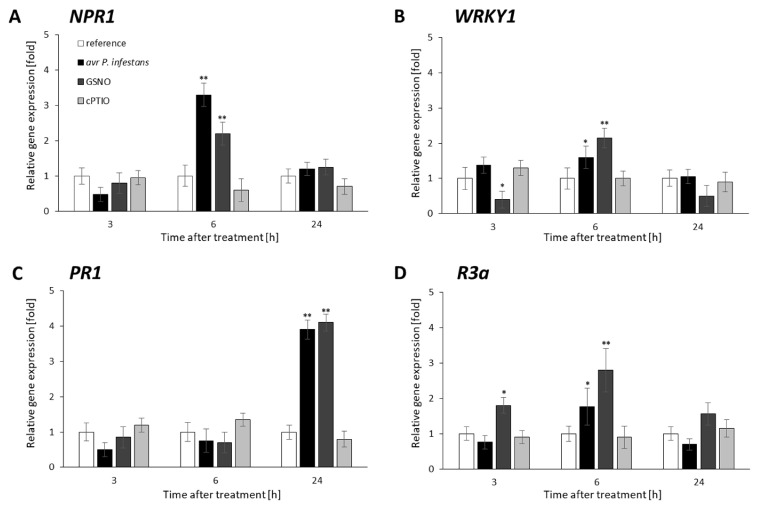
Effects of avr *P. infestans* and GSNO on potato defense genes transcription. RT-qPCR analysis of the *NPR1* (**A**), *WRKY1* (**B**), *PR1* (**C**), and *R3a* gene expression (**D**) was performed at selected time points at 3–24 h after GSNO, cPTIO treatment, or challenge inoculation, respectively. Values represent the means of data ± SD of at least three independent experiments. Asterisks indicate values that differ significantly from water-treated (reference) potato leaves at α < 0.05 (*) and α < 0.01 (**).

First, weanalyzed the transcriptional pattern of essential defense genes and found that *NPR1*, *WRKY1*, and *R3a* peaked mainly at 6 h and *PR1* at 24 h, after pathogen or GSNO treatment (Figure 2A–D). Importantly, the obtained data revealed possible interconnections between controlled NO levels and other NO targets that integrated external cues to internal transcriptional network reprogramming for resistance.

### 2.2. CLF and TrxG Gene Expression under Redox-Dependent Changes

**Figure 3:**
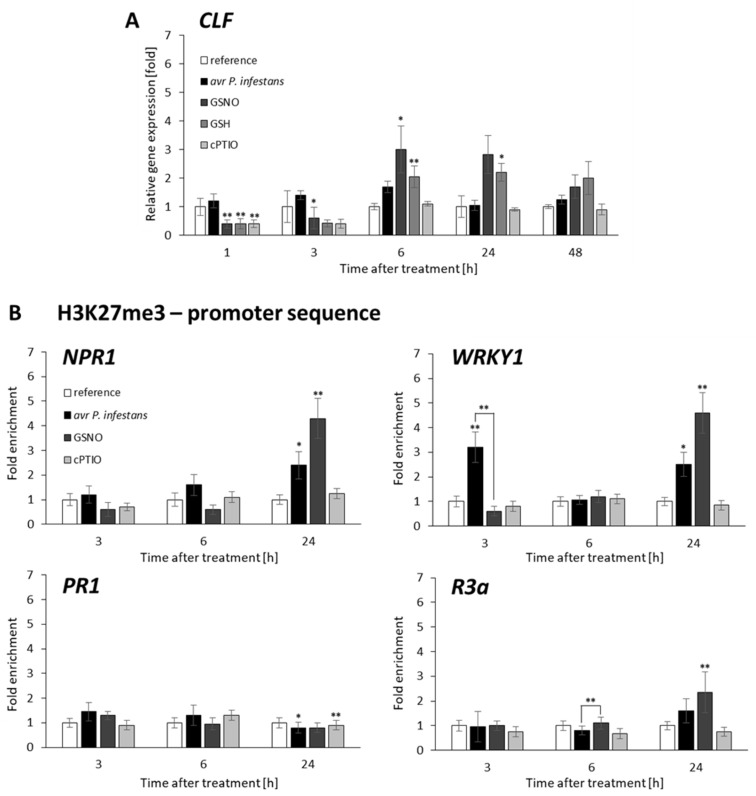
*CLF* methyltransferase expression profile (**A**) and distribution levels of H3K27me3 on the promoter of *NPR1*, *WRKY1*, *PR1*, and *R3a*, respectively (**B**). RT-qPCR gene expression of *CLF* was analyzed in potato leaves (at 1–48 h) after treatment with GSNO, GSH, cPTIO, water, or avr *P. infestans* inoculation, respectively. ChIP–qPCR analyses were performed in potato leaves at selected time points (3–24 h), after treatment with GSNO, GSH, cPTIO, water, or avr *P. infestans* inoculation, respectively. Data are presented as X-fold enrichment [55]. The relative amount of immunoprecipitated chromatin fragments (as determined by real-time PCR) from the above variants of treatment was compared with the reference (arbitrarily set to 1). Each experiment included at least three independent measurements per sample. *P* values for each sample combination were calculated using ANOVA and mean values were compared using the Tukey–Kramer test (α = 0.05 (*) and α = 0.01 (**)).

**Figure 4:**
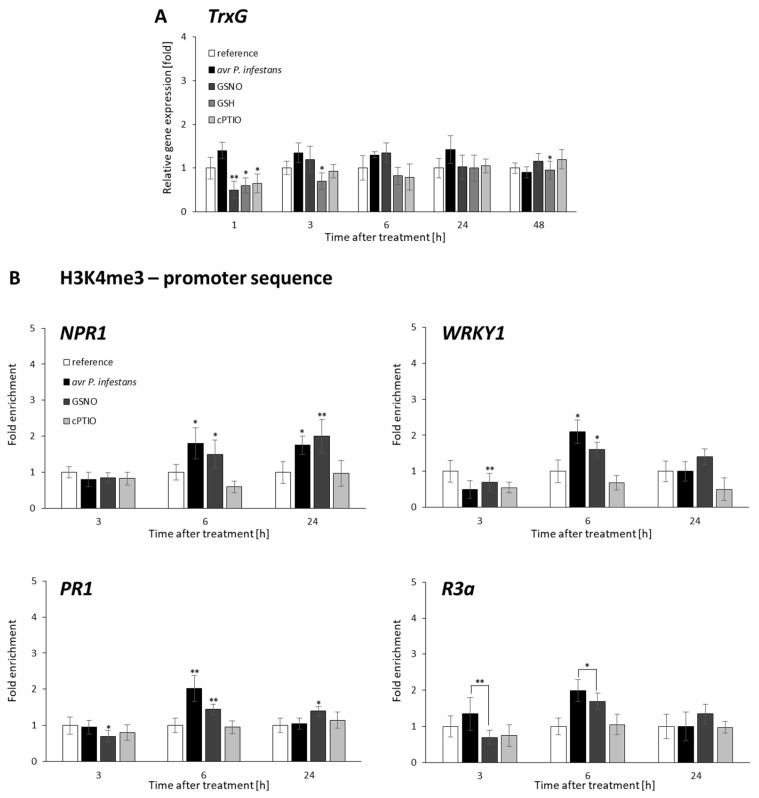
*TrxG* methyltransferase expression profile (**A**) and distribution levels of H3K4me3 on the promoters of *NPR1*, *WRKY1*, *PR1*, and *R3a*, respectively (**B**). RT-qPCR gene expression of *CLF* was analyzed in potato leaves (at 1–48 h) after treatment with GSNO, GSH, cPTIO, water, or avr *P. infestans* inoculation, respectively. ChIP–qPCR analyses were performed in potato leaves at selected time points (3–24 h), after treatment with GSNO, GSH, cPTIO, water, or avr *P. infestans* inoculation, respectively. Data are presented as X-fold enrichment [55]. The relative amount of immunoprecipitated chromatin fragments (as determined by real-time PCR) from the above variants of treatment was compared with the reference (arbitrarily set to 1). Each experiment included at least three independent measurements per sample. *P* values for each sample combination were calculated using ANOVA and mean values were compared using the Tukey–Kramer test (α = 0.05 (*) and α = 0.01 (**)).

To explain how and whether NO bursts affect genes of H3 lysine methyltransferases, we analyzed the accumulation of mRNA transcripts for curly leaf (CLF) and trithorax (TrxG) in response to avr *P. infestans*. (Figure 3A and Figure 4A). Pathogens weakly affected *CLF* gene expression, except for transient stimulation at 6 hpi (Figure 3A). A similar tendency was found in the transcriptional profile for *TrxG*, with no significant higher transcript levels (at 1–24 hpi). (Figure 4A). In turn, GSNO, similarly to GSH treatment, initially decreased (at 1–3 h) *CLF* gene expression, then induced an increase (at 6–24 h), as compared with cPTIO (Figure 3A). *TrxG* gene expression, apart from early (at 1 h) transcript level decline, showed no significant changes in response to GSNO (Figure 4A).

### 2.3. Distribution Status of CLF-Mediated H3K27me3 and TrxG-Mediated H3K4me3 Marks on Stress-Responsive Genes Shows Some Similarities in the Response to Pathogens and GSNO

To investigate the correlation of the transcriptional status with the level of H3K4me3 and H3K27me3 marks on stress-responsive genes after *P. infestans* or GSNO treatment, we used the ChIP–qPCR assay with H3K4me3 and H3K27me3 specific antibodies and primers designed to probe the promoter regions of these genes.

The promoters of *NPR1*, *WRKY1*, and *R3a* exhibited an increase in H3K27me3 levels mainly at a later time point (at 24 h) after both treatments. Only *WRKY1* was also (at 3 h) enriched early in this repressive mark, which decreased (at 6 hpi) after the pathogen challenge (Figure 3B). Notably, a relatively high level of H3K4me3 was noted on the promoter of all stress-responsive genes, mainly at 6 h in response avr *P. infestans* or GSNO (Figure 4B).

The obtained results revealed some similarities between endogenous and exogenous NO sources on the transcriptional pattern of stress-responsive genes linked with the distribution of active mark H3K4me3. After pathogen inoculation, time-dependent enrichment of the H3K4me3 mark on *NPR1*, *WRKY1*, *PR1*, and *R3a* promoters was probably favorable for effectively reinforcing defense gene transcription. Moreover, we concluded that histone lysine methyltransferases activities connected with transient H3K4me3 or H3K27me3 mark deposition on defense genes could operate independently of *TrxG* or *CLF* transcription.

### 2.4. P. infestans and GSNO Modify PRMT5 Activity and Expression

**Figure 5:**
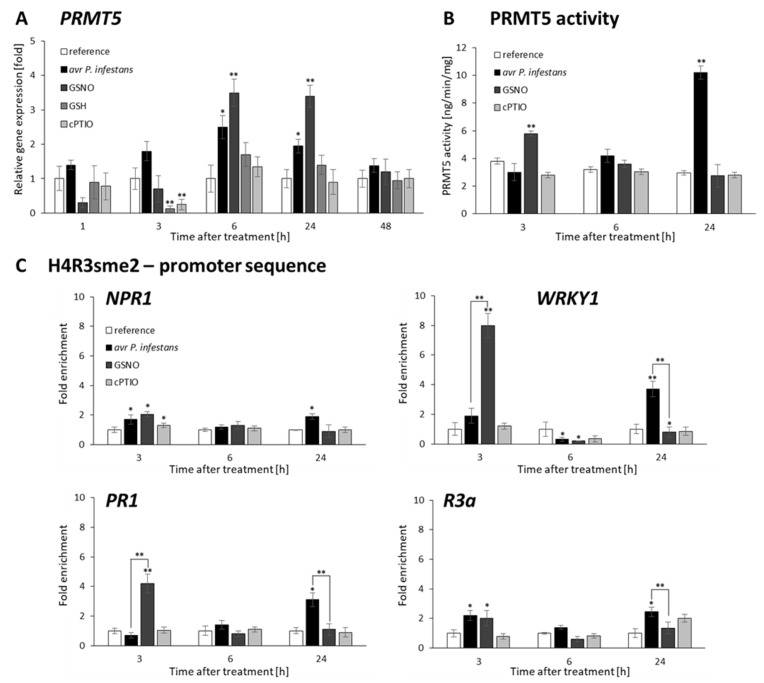
NO-mediated changes in histone arginine methylation *PRMT5* expression (**A**), PRMT5 activity (**B**) distribution levels of H4R3sme2 on the promoter of *NPR1*, *WRKY1*, *PR1*, and *R3a*, respectively (**C**). RT-qPCR and ELISA tests were performed in potato leaves (at 1–48 h) after treatment with GSNO, GSH, cPTIO, water, or avr *P. infestans* inoculation, respectively. ChIP–qPCR analyses were performed in potato leaves at selected time points (3–24 h), after treatment with GSNO, GSH, cPTIO, water, or avr *P. infestans* inoculation, respectively. Data are presented as X-fold enrichment [55]. The relative amounts of immunoprecipitated chromatin fragments (as determined by real-time PCR) from the above variants of treatment were compared with the reference (arbitrarily set to 1). Each experiment included at least three independent measurements per sample. *P* values for each sample combination were calculated using ANOVA and mean values were compared using the Tukey–Kramer test (α = 0.05 (*) and α = 0.01 (**)).

Apart from lysine methylation, we attempted to analyze specific histone modifications in the form of symmetric dimethylation at arginine residue R on histone H4 (H4R3sme2) mediated by PRMT5. Interestingly, the *PRMT5* gene expression displayed a similar trend in transcriptional pattern peaking at 6–24 h after GSNO treatment or avr *P. infestans* inoculation (Figure 5A). However, we did not find a direct coincidence between PRMT5 activity and gene expression after both treatments (Figure 5B). The GSNO induced early upregulation (at 3 h) and pathogen elicited later upregulation (at 24 hpi) of PRMT5 activity. Additional controls, such as GSH or cPTIO treatment, caused no significant differences in PRMT5 activity or gene expression in the following hours compared with GSNO.

### 2.5. PRMT5 Affects Defense Genes Expression by Transient Deposition of the H4R3sme2 Mark

To further combine PRMT5 activity with PRMT5-mediated histone modification, we analyzed the H4R3sme2 mark level on selected genes. Significant enrichment of H4R3sme2 was found in the promoter region of *WRKY1* (eightfold increase) and *PR1* (fourfold increase) early (3 h) after GSNO treatment (Figure 5C). A similarly enhanced level (approximately twofold increase) of this repressive mark was also observed in the promoter region of the *R3a* and *NPR1* genes after both treatments. Next, the same regions of genes promoter were analyzed at later time points and found that H4R3sme2 levels drastically and temporarily decreased at 6 h. Interestingly, the resurgence of this repressive mark level was observed later (at 24 h), but only after pathogen inoculation.

Data indicate that PRMT5 selectively mediated H4R3sme2 and labeled promoters of stress-responsive genes in a time-dependent manner. The lowering PRMT5 activity (at 6 h) resulted in the reduced occupancy of repressive H4R3sme2 on the promoter of genes, at the same time point in response to GSNO or the pathogen. In turn, there was a weak association between PRMT5 activity and *PRMT5* gene expression changes, suggesting a putative involvement of PRMT5 in other metabolic processes related to potato immunity to *P. infestans*.

### 2.6. PRMT5 Contributes to the Hypersensitive Response of Potato to Avr P. infestans

**Figure 6:**
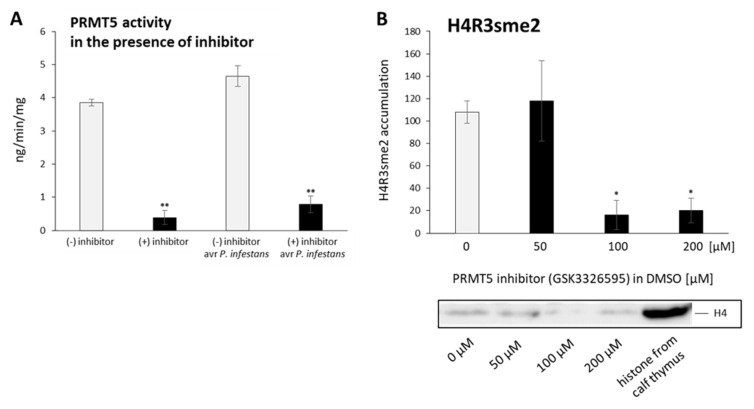
PRMT5 inhibitor (GSK3326595) drastically reduces PRMT5 activity (**A**) and causes a dose-dependent decrease in the total amount of H4R3sme2-marked histone proteins (**B**). ELISA of PRMT5 histone protein activity was performed using potato leaves after the following separate treatments: DMSO; inhibitor in DMSO; DMSO followed by avr *P. infestans* (6 hpi) or inhibitor in DMSO followed by avr *P. infestans* (6 hpi), respectively. For Western blot analysis, potato leaves were treated with increasing concentrations (50, 100, 200 µM) of GSK3326595 in DMSO or DMSO as the control. Total histone proteins were probed with H4R3sme2-specific antibodies and H4 histone from the calf thymus as a loading control. Values represent the means of data ± SD of at least three independent experiments. Asterisks indicate values that differ significantly from DMSO at α < 0.05 (*) and α < 0.01 (**).

To assess whether PRMT5 activity affects the hypersensitive response (HR)-mediated cell death, we applied the PRMT5 inhibitor (GSK3326595), which effectively reduces PRMT5 activity. Thus far, this novel human therapeutic target used as a potent and reversible inhibitor of enzymatic activity of PRMT5 [56] has never been tested on plants. The ELISA confirmed a drastic decrease in PRMT5 activity under the influence of the PRMT5 inhibitor treatment compared with DMSO, used as the control (Figure 6A). After pathogen inoculation, PRMT5 activity slightly increased. Densitometric analysis of Western blot also revealed that the enzymatic inhibitor of PRMT5 (100 and 200 μM) provoked an approximately sixfold decline in total histone proteins marked by H4R3sme2 compared with DMSO (Figure 6B).

Given the importance of PRMT5 activity in numerous cellular processes, we focused on its role in cell damage during hypersensitive potato responses to avr. *P. infestans*. Emerging evidence suggests that NO as a signaling compound together with H_2_O_2_ plays a crucial role in HR-mediated cell death during the ETI response to various pathogens [1,6,17].

**Figure 7:**
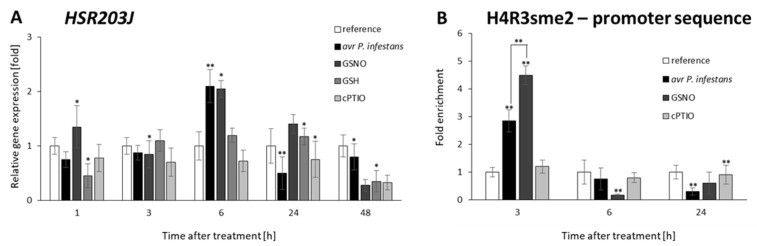
Analysis of *HSR203J* (hypersensitive marker) gene expression (**A**) and time-dependent distribution levels of H4R3sme2 on the promoter of *HSR203J* (**B**). RT-qPCR gene expression of *CLF* was analyzed in potato leaves (at 1–48 h) after treatment with GSNO, GSH, cPTIO, water, or avr *P. infestans* inoculation, respectively. ChIP–qPCR analyses were performed in potato leaves at selected time points (3–24 h), after treatment with GSNO, GSH, cPTIO, water, or avr *P. infestans* inoculation, respectively. Data are presented as X-fold enrichment [55]. The relative amounts of immunoprecipitated chromatin fragments (as determined by real-time PCR) from the above variants of treatment were compared with the reference (arbitrarily set to 1). Each experiment included at least three independent measurements per sample. *P* values for each sample combination were calculated using ANOVA and mean values were compared using the Tukey–Kramer test (α = 0.05 (*) and α = 0.01 (**)).

First, we analyzed the expression of the *HSR203J* gene coding serine hydrolase, which displays an esterase activity. It is well documented that transient intensification of the mRNA transcript accumulation for *HSR203J* is closely associated with the activation of hypersensitive cell death during specific interaction of the *Avr* and *R* genes in *Solanaceae* plants [57,58]. Consistent with these results, we found a solid and time-dependent upregulation of the *HSR203J* gene expression (twofold increase) at 6 h after GSNO exposure or avirulent pathogen inoculation, compared with cPTIO or the healthy leaves, respectively (Figure 7A). Interestingly, the high level (at 3 h) of the repressive H4R3sme2 mark in the *HSR203J* promoter rapidly decreased at 6 h after NO-donor and pathogen treatment, negatively correlated with enhanced *HSR203J* expression at the same time point (Figure 7B).

**Figure 8:**
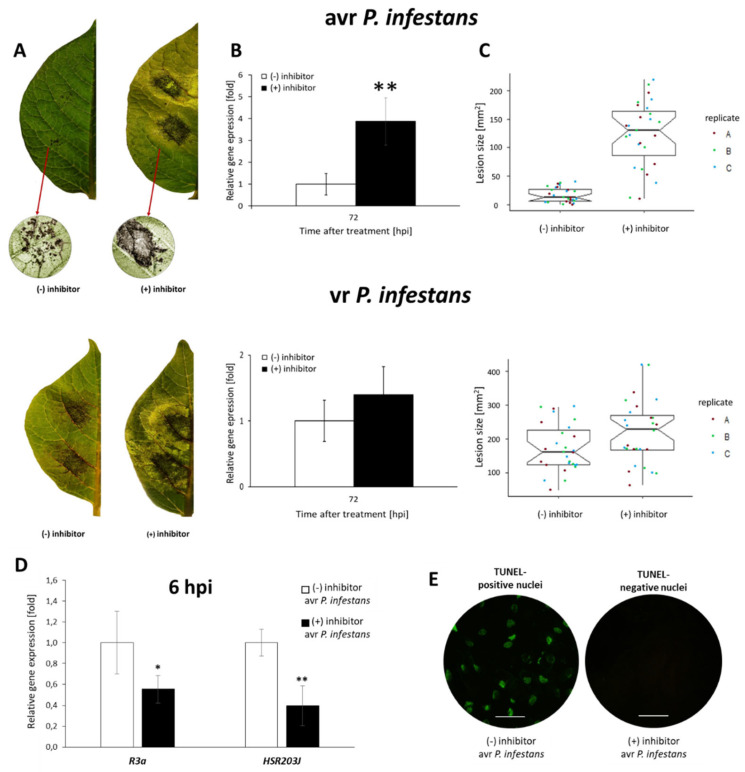
PRMT5 activity is required in potato resistance to avr *P. infestans*. Potato leaf surface (with or without PRMT5 inhibitor) covered by late blight symptoms (**A**), Pitef1 gene expression (**B**) and measurement of lesion size at 72 hpi after challenge inoculation with avirulent or virulent *P. infestans* (MP977) (**C**). Gene expression of R3a and HSR203J (**D**) and TUNEL assay at 6 hpi (**E**). Categorical scatter plots show lesion diameters of twelve inoculated sites from three biological replicates marked with three colors. Gene expression values represent the means of data ± SD of at least three independent experiments, each with at least three biological replicates. Asterisks indicate values that differ significantly from mock-inoculated (water treatment) potato leaves at α < 0.05 (*) and α < 0.01 (**).

Assessing the engagement of PRMT5 in pathogen-induced cell death, we first verified whether PRMT5 downregulation by an enzymatic inhibitor compound might not only cause changes in the transcription of the *R3a* gene, but also investigated how the functional loss of PRMT5 affects local potato immune responses to the pathogen. Based on the disease index assay in potato leaves representing the percentage of leaf area covered by late blight symptoms, we showed that leaves treated with the PRMT5 inhibitor were more susceptible to damage after *P. infestans* inoculation (Figure 8A–C). Thus, the PRMT5 inhibitor, applied before challenge inoculation, caused rapidly developing and highly diffuse disease lesions, in contrast to topically located HR-type lesions on infected leaves without an inhibitor. Moreover, the pharmacological inhibition of PRMT5 also revealed a more significant progression of disease when potato leaves were challenged with virulent *P. infestans*.

To quantify the pathogen biomass in inoculated potato leaves, the expression of the *P. infestans translation elongation factor 1α* (*Pitef1*) gene was measured. A fourfold higher level of *Pitef1* transcription at 72 hpi was observed compared with inoculated plants lacking the PRMT5 inhibitor (Figure 8B). This finding revealed that drastic inhibition of PRMT5 activity counteracted the resistance to late blight. Notably, the PRMT5 inhibitor downregulated *R3a* and *HSR203J* genes and abolished PCD, which was confirmed by TUNEL negative assay (Figure 8D,E).

In conclusion, the obtained data support the hypothesis that PRMT5 contributes to late blight resistance. After a pathogen challenge, a transient decrease in the level of PRMT5-mediated H4R3sme2 plays a critical role in regulating defense responses and co-activating programmed cell death.

## 3. Discussion

### 3.1. Biphasic NO Generation Indirect Reprograms Defense Gene Expression

Nitric oxide is a master regulator of plant immunity; however, knowledge on NO engagement in the epigenetic regulation of defense gene expression remains largely uncharacterized. Pathogens can seriously disturb NO homeostasis and elicit NO overproduction, generally known as the NO-burst. It has been well documented that biphasic NO/H_2_O_2_ influences HR [59,60]. Our results confirmed two waves of NO generation in potatoes inoculated with avr *P. infestans*, mediated by nitrate reductase (NR) activity [61]. Along with NO generation, GSNOR activity engaged in controlling the cellular level of NO/SNOs and GSNO content was upregulated early in response to HR-eliciting *P. infestans*. Previously, the linkage between the absence of AtGSNOR1 and reduced *R* gene-mediated resistance to *PstDC3000* in the *atgsnor1-3* line compared with wild-type *Arabidopsis* was presented by Feechan et al. [62]. In other plants and under stress conditions, it has been demonstrated that GSNOR activity is differentially involved in NO homeostasis and SNOs accumulation [63]. Notably, GSNOR is also present in the cell nucleus compartment, together with GSNO and small nitrosothiols (CysSNO), representing a reservoir and transport form of NO [40,64].

Searching for NO implication in the epigenetic regulation of potato immunity to late blight, we found enrichment of the H3K4me3 mark on the promoter region of *NPR1*, *WRKY1*, *PR1*, and *R3a* after pathogen inoculation, positively correlated in timing to genes expression. In turn, early CLF-mediated H3K27me3 levels did not significantly differ in the promoter of most dedicated genes, except for *WRKY1*. Then, a temporary decrease in H3K27me3 (at 6 h) was observed on the *WRKY1* promoter following pathogen challenge, independently of bivalent chromatin containing H3K4me3 on the same allele, which probably upregulated transcription. H3K4me3 did not preclude the accumulation of H3K27me3 on promoter regions of analyzed genes. The H3K4me3 mark, together with the H3K27me3 on defense-related genes, needs to be tightly balanced for faster inhibition or activation upon pathogen attack. However, it happens that histone modifications associated with specific marker deposition across genomic loci can occur independently of transcriptional activation or repression under stress conditions [65,66,67]. It was also found by Liu et al. [68] that the high level of H3K27me3 at specific dehydration stress-responding genes did not preclude the accumulation of H3K4me3 when the genes were actively transcribed. Generally, the functional consequence between the histone methylation mark and gene expression is a highly complex event, greatly depending on different combinations of histone PTMs, interactions with enhancers, and other histone modifiers that can coincide in response to NO/SNOs during pathogen attack.

However, our data concerning biphasic kinetics of NO burst and defense genes expression revealed that the rather declining phase and low level of NO might be decisive in facilitating the pathogen-induced upregulation of stress genes.

Several reports have revealed the functional role of NO/SNO from bacteria to mammals in direct or indirect epigenetic NO effects on transcription factors, chromatin remodeling enzymes, and histones [17,41,69,70]. As proven, NO epigenetically regulates histone deacetylase (HDAC) through the selective S-nitrosation or tyrosine nitration of HDACs in animals and plants. S-nitrosation of cysteine residues at binding sites on HDACs was found to inhibit enzyme activities and impair their ability to bind DNA in *Arabidopsis* [45,46]. An intricate crosstalk between NO and HDACs has been widely discussed in terms of different physiological and pathophysiological aspects in animals and humans [71,72]. Plant histone H3 and H4 acetylation by targeting and inhibiting histone deacetylases (HDA6/HDA19 complex) in *Arabidopsis* nuclear extracts and protoplasts exposed to GSNO were presented by Mengel et al. [45]. According to Ageeva-Kieferle et al. [47], GSNOR and HDA6 might maintain a tightly controlled balance between the acetylation/deacetylation states of genes involved in various developmental or stress metabolism processes.

Given the documented data concerning NO links with histone acetylation, the study on NO engagement in methyl-lysine or arginine modifications of target chromatin loci of histones H3/H4 is in its infancy in plants. Similar to histone acetylation, histone methylation is not a permanent modification, and both of these modifications often cooperate or antagonize. Although it has recently been documented by Rudolf et al. [48] that the GSNOR function is also required for balancing the methylation index between the prominent methyl donor, S-adenosylmethionine (SAM), for histone or DNA methylation and S-adenosylhomocysteine (SAH), its byproduct (the SAM/SAH ratio). Finally, the authors postulated that GSNOR1 plays a crucial role in regulating methylation processes and stress-responsive gene expression [48].

In mammals, in contrast to plants, advanced studies have been carried out for many years, providing essential insights on how NO can regulate the transcription of genes by changing global acetylation and methylation levels of histones [69,73,74,75].

#### 3.1.1. NO and PRMT5 Activity Are Required to Integrate the Transcription of Defense Genes

PRMT5 (SKB1) catalyzes H4R3sme2 and usually functions in repressing target gene expression; however, its specific regulation under stress conditions remains unclear in plants. The methylation of arginine residues governed by PRMT5 is less stable than the methylation of lysine PTMs, with a half-life ranging from several hours to days [69]. According to Fan et al. [33], PRMT5/SKB1 lays down H4R3sme2, a repressive mark, playing a role in sensing environmental cues. The genome-wide analysis of wild-type and *prmt5* mutants did not support the idea that PRMT5 specifically acts as a transcriptional repressor, as previously suggested based on the analysis of several genes [76]. The authors found that 2604 genes were over-expressed and 3075 were under-expressed in *prmt5* mutants under non-stress conditions as compared with wild-type *Arabidopsis*.

Our results indicate that in response to the pathogen, lowering PRMT5 activity (at 6 hpi) was correlated with the declining level of H4R3sme2 on the defense gene promoter, which enhanced the transcription of these genes. Notably, a decreased level of the repressive H4R3sme2 mark occurred concurrently with an increased level of active H3K4me3 marks on the same promoter regions, revealing crosstalk between lysine and arginine methyltransferases. The biphasic NO production shifted down at 6 hpi was probably decisive for switchable changes in histone marks on the defense gene promoters.

Data presented by Hu et al. [37] documented that NO positively regulates PRMT5 activity by S-nitrosation at Cys-125 in response to salt stress. The *gsnor1-3* mutant, with significantly higher levels of GSNO and S-nitrosothiols, was more resistant to stress than wild-type *Arabidopsis*. The authors observed no changes in the level of H4R3sme2 in total protein extracts under NaCl conditions, and linked stress-induced PRMT5 activity with the pre-mRNA splicing machinery associated with stress-related genes.

Generally, prior studies have revealed that under salt stress or ABA treatment, a high H4R3sme2 level decreased due to PRMT5/SKB1 disassociating from chromatin, induced the expression of *FLC* with stress-responsive genes, and improved the efficiency of the pre-mRNA splicing process [32]. In turn, when plants were subjected to low temperature (vernalization) or short/long days, the levels of H4R3sme2 associated with the chromatin of *FLOWERING LOCUS C* (*FLC*) increased or decreased, depending on the treatment affecting the flowering program [29,30].

Moreover, it was previously documented that PRMT5/SKB1-mediated H4R3sme2 on the promoter of the *Ib* subgroup *bHLH* transcription factor genes could be involved in the regulation of iron homeostasis in *Arabidopsis* [33]. Under iron deficiency conditions, the level of H4R3sme2 associated with *bHLH* decreased, resulting in the reduced transcriptional repression of genes and enhanced iron uptake.

It is known that NO plays a crucial role in iron homeostasis in plants [32]. Apart from NO engagement in regulating genes related to iron acquisition, NO reacts with iron-producing dinitrosyl iron complexes (DNICs). In animals, it is well documented that DNICs can indirectly inhibit the Jumonji C (JMJC) domain-containing histone demethylases. NO can also directly inhibit the catalytic activities of these enzymes by binding to the non-heme iron in the catalytic pocket. Thus, it was found by Hickok et al. [77] that human JMJC domain-containing histone demethylase KDM3A was highly sensitive to inhibition by NO.

There is no doubt that a peculiar crosstalk between methyltransferases/demethylases modifying histone patterns exists under stress conditions; however, there is no information on NO engagement in inhibiting JMJC domain-containing histone demethylases in plants. It was proposed that GSNO reductase (GSNOR) activity regulating the intracellular level of NO indirectly contributes to demethylation processes in *Arabidopsis* [48]. This finding aligns with our result, suggesting that GSNOR might control the NO level during biotic stress and indirectly affects histone methylation in orchestrating defense responses in potato.

Recently, intriguing evidence has been presented, indicating that NO modulates the selective autophagic degradation of GSNOR1 by S-nitrosation at Cys-10 and positively regulates responses under hypoxia [15].

#### 3.1.2. NO Cooperates with PRMT5 in the Regulation of Hypersensitive Cell Death and Potato Resistance to Late Blight

AtPRMT5 has been found to play an essential role in regulating plant vegetative growth, flowering time, and various other cellular and biological processes, including apoptosis [28,29,31,32,34,37,78,79]. One of the most typical features of ETI response is the rapid dying of host plant cells at the site of infection within hours following pathogen contact; this process of programmed cell death is known as the hypersensitive response [80]. Our data revealed that the pharmacological inhibitor of PRMT5 downregulated *R3a* gene expression and abolished HR-type resistance, evidenced by TUNEL negative assay.

Moreover, the *HSR203J* gene, as an HR marker, was activated at the same time points after GSNO or avr *P. infestans* inoculation. Initially, the PRMT5-mediated high level of H4R3sme2 on the promoter of *HSR203J* drastically decreased, correlated with upregulation of the HSR203J transcript and triggering cell death.

The *hsr203j* gene is a valuable marker of HR mediated by *R/avr* genes activated in tomato against *Cladosporium fulvum* (*Cf-9/avr9*), tobacco against *Ralstonia solanacearum*, and in similar processes in other members of *Solanaceae* [57]. Significantly, *hsr203j* is not expressed during leaf senescence, and four W boxes with MYB binding sites have been identified in the enhancer region of the *HSR203J* promoter [58]. It remains unclear whether PRMT5-mediated H4R3sme2 directly regulates the transcriptional activity of *HSR203J* in response to cell death provoked by an avirulent pathogen. *HSR203J* might be the target for *WRKY1* or other transcription factors recognizing the W-box present in the promoter of various pathogen-related genes involved in defense responses. Our findings suggest a causal link between *WRKY1* and *HSR203J* in response to avr *P. infestans*.

Moreover, it was previously documented that *StWRKY1* participates in other defense pathways, including regulating phenylpropanoid metabolite gene expression, strengthening the secondary cell wall, and enhancing potato resistance to *P. infestans* [81,82]. Modifying the host cell wall to the plasma membrane continuum is critical for sensing and inducing several interlinked and independent defense signaling compounds, e.g., ROS/NO burst and cell death [83].

Previously Li et al. [34] suggested that *PRMT5/SKB1*-deficient root stem cells of the *skb1* mutant were more sensitive to DNA damage caused by a genotoxic agent (methyl methanesulfonate, MMS). Numerous animal studies have suggested that the dysregulation of H4R3sme2 through downregulation of the PRMT5 protein level modifies the expression of target genes essential in cancer survival [84] and can selectively diversify the proteome via alternative splicing [85].

Many targets of PRMT5 in living cells have been identified until now. PRMT5/SKB1 might interact with different transcription factors and chromatin-modifying enzymes and mediates the methylation of nuclear proteins functioning as a co-repressor and co-activator during differentiation or apoptosis [79,86,87,88,89,90].

In conclusion, our findings postulate that biphasic NO production, downregulated by GSNOR activity, is required to reprogram the transcriptional network of defense genes. A decreased level of the repressive H4R3sme2 mark occurred (at 6 hpi) concurrently with an increased level of the active H3K4me3 mark on the same promoter regions, revealing crosstalk between lysine and arginine methyltransferases. A time-dependent reduction in the level of PRMT5-mediated H4R3sme2 on the *R3a* and *HSR203J* promoters enhanced their expression and triggered HR-type resistance to avr *P. infestans*. Future studies should be performed to gain greater insight into the epigenetic mechanism influencing pre-mRNA splicing machinery, by which PRMT5 regulates cell death in potato exposed to avr *P. infestans*.

## 4. Materials and Methods

### 4.1. Plant Material

All experiments were conducted on potato plant *Solanum tuberosum* L. cultivar Sarpo Mira (carrying the R genes: *R3a*, *R3b*, *R4*, *Rpi-Smira1*, and *Rpi-Smira2*), which is highly resistant to avr *P. infestans*. In vitro potato seedlings came from the Potato Genebank (Plant Breeding and Acclimatization Institute IHAR-PIB, Bonin, Poland). Plants propagated from in vitro nodal cuttings were grown for 4 weeks in sterile MS medium (Duchefa Biochemie B.V. Haarlem, The Netherlands) containing 2% (*w*/*v*) sucrose and 10% agar. Afterwards, plants were transplanted to sterile soil (universal substrate consisting of natural peat, WOKAS SA, Łosice, Poland) and grown to the leaf stage in a phytochamber with 16 h of light (180 μmol m^−2^ s^−1^), FLUORA L18W/77, and L58W/77, OSRAM, Germany) at 18 ± 2 °C and 60% humidity.

### 4.2. Pathogen Culture and Inoculation

The avr *Phytophthora infestans* Mont. de Bary isolate MP946 (A1 mating type, race 1.3.4.7.10.11) was kindly supplied by the Plant Breeding and Acclimatization Institute collection Research Division in Młochów, Poland. The pathogen grew for 3 weeks on a pea medium, and was subsequently passaged through tubers at least twice. Inoculated slices of tubers were incubated for 7–14 days at 16 °C in the dark. The sporangia of *P. infestans* were obtained by collecting the aerial mycelium, rinsed with cold distilled water, passed through a sterile sieve, and adjusted to a concentration of 2.5 × 10^5^ sporangia per 1 mL using a hemocytometer. Then, the sporangia were incubated at 4 °C for 1 h to release the zoospores. Potato plants were inoculated by spraying leaves with a zoospore suspension and kept overnight at 18 °C and 90% humidity on moist blotting paper in a plastic box covered with glass. Afterward, inoculated and control leaves were sprayed with distilled water and transferred to a phytochamber. Samples were collected at 3, 6, 24, and 48 h post-inoculation (hpi).

### 4.3. Molecular Quantification of Pathogen

The *P. infestans translation elongation factor 1α* (*Pitef1*) gene was expressed in inoculated potato leaves, as confirmed by RT-qPCR analysis. The level of *Pitef1* transcription was calculated for *18sRNA* and *ef1α* gene expression [91].

### 4.4. Assessment of Disease Development

For the point inoculation experiment, 20 µL drops of the zoospore suspension were applied on the abaxial leaf surface, and leaves were kept at 100% humidity in a growth chamber. Lesion diameters (mm^2^) of 12 infection sites from 3 independent biological replicates on potato leaves were measured at 72 hpi using the Adobe PHOTOSHOP CS5 (12.0) program. The mean area of a diseased spot was calculated to the area of transferred inoculum droplets.

### 4.5. NO Donor and Scavenger Treatment

The third or fourth compound leaves from the base of intact plants were treated by spraying with 250 µM GSNO (Sigma–Aldrich), then closed in an air-tight plastic chamber and exposed to light. To evaluate the effect of NO elimination in GSNO-treated potato, the leaves were also treated with a specific NO scavenger, cPTIO (Sigma–Aldrich), at 200 µM. Moreover, 250 µM of GSH (Sigma–Aldrich) was applied as a reducing compound in contrast to oxidizing GSNO under physiological conditions. GSH possesses a similar structure to GSNO, but cannot generate NO. The leaves were treated by spraying with 5 mL of the solution. Samples were collected at 3, 6, 24, and 48 h after treatment.

### 4.6. PRMT5 Inhibitor Treatment

GSK3326595 (MedChemExpress, HY-101563) is a potent, reversible inhibitor of protein arginine N-methyltransferase. Leaves were sprayed with 5 mL GSK3326595 (50, 100, or 200 μM) in 1% DMSO, or with an equal volume of 1% DMSO as the control. After 12 h of incubation, leaves were dried and inoculated with avr *P. infestans* as described above. Samples were collected at 3 h and 6 h after inoculation.

### 4.7. NO Detection and Quantification by the Electrochemical Method

Electrochemical monitoring of NO emissions from 250 µM GSNO under continuous illumination intensity (180 µmol photons m^−2^ s^−1^; starting temperature 20 °C; final temperature 26 °C) was performed as described previously by Floryszak-Wieczorek et al. [54].

### 4.8. Measurement of Nitric Oxide Generation

Nitric oxide generation was quantitatively measured using DAF-2DA (Calbiochem). Potato leaf samples (0.1 g) were incubated in the dark for 1 h at 25 °C in a mixture containing 10 μM DAF-2DA in 10 mM Tris–HCl buffer (pH 7.2). After incubation, the probes were transferred into 24-well plates (1 mL per well). Fluorescence in the reaction was measured using a spectrofluorometer (Fluorescence Spectrometer Perkin Elmer LS50B, United Kingdom) at 495 nm excitation and 515 nm emission filters. Fluorescence was expressed as arbitrary fluorescence units.

### 4.9. S-Nitrosoglutathione Reductase [EC 1.2.1.46]

The GSNOR activity was assayed according to the procedure proposed by Barroso et al. [92], with modifications as described by Janus et al. [93]. Fresh leaves (0.5 g) were homogenized in 0.1 M Tris–HCl buffer, pH 7.5 (1:4 *w*/*v*) containing 0.2% Triton X-100 (*v*/*v*), 10% glycerol (*v*/*v*), 0.1 mM EDTA, 2 mM DTT at 4 °C and centrifuged at 27,000× *g* for 25 min. The supernatant was passed through Sephadex G-25 gel filtration columns (Illustra NAP-10, GE Healthcare), then immediately through Amicon Ultra 3 K Filters (Millipore) and served as the enzyme extract. The 1 mL assay reaction mixture contained 0.5 mM EDTA, 0.2 mM NADH, 0.4 mM GSNO and 30 μL enzyme extract in 25 mM Tris–HCl buffer, pH 8.0. The reaction was held at 25 °C and initiated with NO (Sigma Aldrich). NADH oxidation was determined at 340 nm, and rates of NADH consumed per minute were calculated using an extinction coefficient of 6220 M^−1^ × cm^−1^.

### 4.10. TUNEL Assay

The TUNEL assay measures DNA fragmentation using the terminal deoxynucleotidyl transferase (TdT)-mediated deoxyuridine triphosphate (dUTP) nick end labeling method, which involves the TdT-mediated addition of fluorescein-12-dUTP to the 30 OH ends of fragmented DNA. The samples were studied using a TUNEL fluorescein kit (Roche; United States), in accordance with Floryszak-Wieczorek and Arasimowicz-Jelonek [94], and examined using a fluorescence microscope (Axiostar plus, Carl Zeiss, Germany) equipped with a digital camera, with excitation at 488 nm and emission at 515 nm. Experiments were repeated four times with ten slides per treatment. A region of 100 cells from at least 5 randomly selected slices in each treatment was counted and statistically analyzed.

### 4.11. Gene Expression Analysis

Potato leaves were frozen in liquid nitrogen and stored at −80 °C before use. RNA was isolated from frozen leaf tissue (150 mg) with TriReagent (Sigma, USA). The obtained RNA was then purified using a deoxyribonuclease kit (Sigma, USA). Reverse transcription of 1 µg of RNA for each experimental variant was performed using a reverse transcription kit (Thermo Fisher Scientific, USA). RT-qPCR analysis was performed on a PikoReal Thermocycler (Thermo Fisher Scientific, USA) under the following conditions: 10 min at 95 °C, followed by 45 cycles of 12 s at 95 °C, 30 s at the annealing temperature for each specific primer (Supplementary Tables S1 and S2) and 30 s at 72 °C. The reaction mixture contained 0.1 μM of each primer, 1 μL of 5 × diluted cDNA, 10 μL of the Power SYBR Green PCR Master Mix (Applied Biosystems, USA) and DEPC-treated water to a total volume of 20 μL. Primers for the studied genes were designed using the Primer-blast program available from the NCBI (National Center of Biotechnology Information, USA) and PGSC (Potato Genome Sequencing Consortium) databases. The primers designed and used in this study are listed in Supplementary Table S1. The obtained data were normalized to elongation factors *ef1α* (AB061263) and *18S rRNA* (X67238). Ct values were determined using the Real-time PCR Miner [95], and relative gene expression was calculated using efficiency corrected computational models proposed by Pfaffl [96] and Tichopad et al. [97].

### 4.12. Chromatin Immunoprecipitation Assay

The chromatin immunoprecipitation assay (ChIP) was carried out as described by Haring et al. [98] and Komar et al. [55]: 2 g of potato leaves was cross-linked by vacuum infiltration in a crosslinking buffer with 1% formaldehyde and then frozen at −80°C. The next step was chromatin isolation, performed according to an existing protocol [98], with some modifications. Samples were ground in liquid nitrogen, resuspended, and incubated in nuclei isolation buffer, and after centrifugation, resuspended in nuclei lysis buffer. Then, the samples were sonicated on ice for 30 × 30 s at 30% of power until DNA fragments of 250–750 nt were obtained. After sonication, an input sample (50–100 μL) was collected from the solution to check the quality of the sample on an agarose gel. The remaining solution was separated into the test sample (to which the antibody of interest was added: H3K4me3 (EMD Millipore; cat.-no. 07-473), H3K27me3 (EMD Millipore; cat.-no. 07-449), or H4R3sme2 (Abcam; cat.-no. ab5823) and the control sample (to which IgG was added). The next day, 30 μL of magnetic beads (PureProteome Protein A/G Mix, Millipore) was added, and the samples were incubated for at least 2 h. After incubation, the samples were washed and decrosslinked overnight with 300 mM NaCl and 1% SDS at 65 °C with shaking. The next step consisted of incubating probes with proteinase K (20 mg/mL) to digest proteins. Then, the samples were subjected to DNA isolation with a phenol/chloroform/isoamyl alcohol mixture (25:24:1). The last step was to check the number of binding sites in the immunoprecipitated DNA using the RT-qPCR method. The reaction mixture contained 0.1 μM of each primer, 2–5 μL of purified DNA, 10 μL of Power SYBR Green PCR Master Mix (Applied Biosystems), and DEPC-treated water, to a total volume of 20 μL. The specificity of the reaction was confirmed by the presence of one peak in the melting curve analysis. Primers for the genes of interest (*NPR1*, *WRKY1*, *PR1*, *R3a*, and *HSR203J*) were designed with Primer3 Output Software (Supplementary Table S2). Data were analyzed by the fold enrichment method [99]. For this purpose, the raw Ct value of each sample was subtracted from the raw Ct value of the control (IgG) corresponding to that sample (ΔCt = Ct _(sample)_ − Ct _(control, IgG)_). The enrichment was calculated using the following formula: Fold enrichment = 2^−ΔCt^.

Samples were taken at 3, 6, and 24 h after treatment with GSH (250 μM), GSNO (250 μM), and cPTIO (200 μM) and after avr *P. infestans* inoculation. The relative amounts of immunoprecipitated chromatin fragments (as determined by real-time PCR) from the above treatment variants were compared with the reference (arbitrarily set to 1). The reference (leaves sprayed with water) was taken at each time point.

Each experiment included at least three independent measurements per sample. The *P* values for each sample combination were calculated using ANOVA. The Tukey–Kramer test was used to compare the mean values (α = 0.05 (*), and α = 0.01 (**)).

### 4.13. ELISA Test for PRMT5 Activity

The level of PRMT5 activity was determined using an Epigenase^TM^ PRMT5 Methyltransferase (Type II-Specific) Activity/Inhibition Assay Kit (Epigentek). Standard curves were generated using standards supplied by the manufacturer (H4R3), with a linear detection range of 0.1–2 ng of the methylated product. Input materials used in this procedure were nuclear extracts of 10 μg. Absorbance was measured using a Tecan Infinite M50 plate reader (ThermoFisher Scientific) at 450 nm (with the reference wavelength of 655 nm).

### 4.14. Histone-Enriched Protein Isolation

Histone-enriched protein was isolated from *S. tuberosum* ‘Sarpo Mira’ leaves, as described by Moehs et al. [100]. Namely, 1 g of leaves was homogenized in 7 mL of the homogenization buffer containing 10 mM Tris HCl pH 8.0, 0.4 M sucrose, 10 mM MgCl_2_, 20 mM β-mercaptoethanol, 0.1 mM phenylmethylsulphonyl fluoride (PMSF). Homogenate was filtered through four layers of miracloth and centrifuged at 9300× *g* for 10 min at 2 °C. The protein pellet was resuspended in 7 mL of the homogenization buffer enriched in 1% Triton X-100 and centrifuged at 9300× *g* for 10 min at 2 °C. The protein pellet was then resuspended in 5 mL of 0.4 M H_2_SO_4_, and overnight extraction at 4 °C (shaking) was performed. The extract was centrifugated at 30,400× *g* for 30 min at 2 °C, and histone-enriched protein was precipitated from the supernatant by adding 3.5 volumes of acetone and overnight incubation at −20 °C. The protein was pelleted at 32,100× *g* for 30 min at 2 °C, dried, and dissolved in 0.2 mL of the buffer containing 0.01 M HCl, 8 M urea, and 0.5 M β-mercaptoethanol. The protein concentration was measured by the Bradford assay [101].

### 4.15. Immunoblot Analysis

Equal amounts (5 µg) of histone-enriched protein were separated by standard SDS-PAGE in 15% polyacrylamide gels, and electrotransferred on a PVDF membrane immunostained with antibodies against H4 symmetric dimethyl Arg 3 (Abcam; catalog number ab5823) applied in a concentration of 0.5 µg/mL. According to standard procedures, signals were visualized using the chemiluminescence method and quantified using Image Lab™ software (Bio-Rad). Statistical significance of the differences in signal intensity was analyzed using the Student’s *t*-test at α < 0.05 (*) and α < 0.01 (**).

### 4.16. Statistical Analysis

All the experiments included three independent experiments in at least three replications. For each experiment, the means of the obtained values were calculated along with standard deviations. Analysis of variance was conducted, and the least significant differences (LSDs) between means were determined using Tukey’s test at the levels of significance α = 0.05 (*), and 0.01 (**). Statistical analyses were performed using Microsoft Excel 2016 and R statistical software (version 4.1.2).

## Data Availability

Not applicable.

## Supplementary Materials

The following supporting information can be downloaded at: https://www.mdpi.com/article/10.3390/ijms23074051/s1.

## Abbreviations

CLF	curly leaf
cPTIO	carboxy-PTIO
DNICs	dinitrosyl-iron complexes
ETI	effector-triggered immunity
GSH	glutathione
GSK3326595	inhibitor of protein arginine N-methyltransferase
GSNO	S-nitrosoglutathione
GSNOR	S-nitrosoglutathione reductase
H4R3sme2	symmetricdi methylation atthearginine residue Ron histoneH4
H3K27me3	trimethylation of histone H3lysine27
H3K4me3	trimethylation of histone H3lysine4
HDACs	histone deacetylases
HMTs	methyltransferases
HR	hypersensitive response
KDMs	lysine demethylases
NR	nitrate reductase
NO	nitricoxide
ONOO¯	peroxynitrite
PAMP	pathogen-associated molecular pattern
PR	pathogenesis-related proteins
PRC	polycomb repressor complex
PRMT5	protein arginine symmetric methyl transferase5
PTI	PAMP-triggered immunity
PTMs	post-translational protein modifications
RdDM	RNA-directed DNA methylation
ROS	reactive oxygen species
siRNA	small non-coding interfering RNA
SKB1	kinasebindingprotein1
TrxG	trithorax
