# Supplementary material for: Nitric Oxide Implication in Potato Immunity to Phytophthora infestans via Modifications of Histone H3/H4 Methylation Patterns on Defense Genes
Source: Int J Mol Sci. 2022 Apr 6;23(7):4051. doi: 10.3390/ijms23074051 (PMC8999698; doi:10.3390/ijms23074051)
Supplement: Supplementary file 1 [file ijms-23-04051-s001.zip › ijms-1660623-supplementary.pdf]

Supplementary Table S1. List of designed primers

Gene	Sequence (5’->3’)		Accession Number	Tm [°C]
	Forward	Reverse		
EF	ATTGGAAACGGATATGCTCCA	TCCTTACCTGAACGCCTGTCA	AB061263	53
18s rRNA	GGGCATTCGTATTTCATAGTC AGAG	CGGTTCTTGATTAATGAAAACAT CCT	X67238	60
CLF	TCCTCAGTGTGCTAGTCATGG	ATGCTTCCTACCTGGCATCTG	XM_006361673	54
HSR203J	AGGAGCTTAAATTGCCGCCT	TCCCACGCCGTTGTTTATCA	PGSC0003DMG400021128	53
NPR1	GCTGAACTTGGTCTACAAGG	TCATCGAGAGTAGTATGCC	PGSC0003DMG400010635	53
Pitef1	ATGACTCGCCTCGGTGATTA	TCCACACACACAAAGTGCATCA	XM_0029 04671.1	60
PR1	AAGACTATCTTGCGGTTCAC	TCTCCTGCACCTGAATGAA	PGSC0003DMG400005110	54
PRMT5	GGAACAACAACCCCTCGTCAC	GGAAGACAATTCGGTGCATAAAT AA	PGSC0003DMG401003285	60
R3a	AGCCAACTGGTGAGATTAGT	ATCCTGTACCCACAATTTGC	PGSC0003DMG400009455	53
TrxG	CAACTTGCGTGTTATTGTGG	ATACAGCTTTGCCATTCGTT	XR_368704	53
WRKY1	TTTCTTCAGCATCATCGTC	TTCCATGCTTGCCATTAGTC	PGSC0003DMT400056725	54

Supplementary Table S2. List of designed primers for ChIP-qPCR (promotor sequence) analyses

Gene	Sequence (5'->3')		Accession Number	Tm [°C]	Promotor region sequence +500bp
	Forward	Reverse			
HSR203J	AGGAGCT<br>TAAATTG<br>CCGCCT<br><br>Start: 40<br>Stop: 61	TCCCACG<br>CCGTTGT<br>TTATCA<br><br>Start: 135<br>Stop: 114	PGSC0003DMG4000<br>21128	56	gaagatacaaagagttttacaaaaatttggactttagatagtgggaagagtaaaggcatt<br>gacagatcattttctacacaatttcctaagtgggtcattccacttatagattttcgtcca<br>catttacaaactggaatataagaaagactttgttaagatccaaataataggacgttataa<br>cgagagttactctctctgtccaacaatacttatttaatgtattaaaaatagttgtttaat<br>gatacgtgtttactttatgaagttcattgataattttacacttacttcctaatttactct<br>tatcattaattatagacattttctcattatatttttcaagatattttatttattatattt<br>aaagggtgatatgataaaattatccttctatttatagtttcttaaagggtgtgtgatgtg<br>ttaagtcaataatggataaattagataatacagaaaaatattctaagagaatcataattt<br>ttttatatgattttaatcact
NPR1	GCTGAAC<br>TTGGTCT<br>ACAAGG<br><br>Start: 52<br>Stop: 74	TCATCGA<br>GAGTAGT<br>ATGCC<br><br>Start: 148<br>Stop: 129	PGSC0003DMG4000<br>10635	55	tcacattatttgttaatttcattaattgtatcctctttttctctagttttgctatgcct<br>tttatgtgagtccaagatctatcgaaaataacatctctatttcataagataaaaataagg<br>ttgcgtataactactctcctcgcatcctatttgtgaagtcacactgaatatgcatgttgt<br>tcaaatagtaactaaaacaaataaattaaaatagagagtatggaagtcaataattagtaa<br>tcgtgctatttaatttctctttccatattatttgttggtgctgctgatattttctatcgt<br>gtttccttttattattttatctgagccaagagtatatcgaaaatattctctcttaacttc

					aaataagactgcatatacatcactctttcaagatctcactcgtgtaatttcattaaatat<br>gttattgttacttatattcccttttttcttttgtttaaatacctcagccgcccagaagaac<br>catccatattgctccaccctc
PR1	AAGACTA TCTTGCG GTTCAC<br><br>Start: 119<br>Stop: 139	TCTCCTG CACCTGA ATGAA<br><br>Start: 192<br>Stop: 170	PGSC0003DMG4000 05110	57	gacttgactcagaaaacatagagggatcatttaaacaatataaaaactttttaaaaaaaa<br>gaacaaattaaattgctcgaatattgtttactagtagtagtatatcctgtggttaaaatt<br>tcttgcagaaggcagaacaaaatttaattgtccaatcaattatatattctcaccaaatcc<br>tacgtttcatcttcttttcattaattatatagtactcctagtaacggataatagtaatgg<br>taattagggagttcaatatatatagtgaaacatcaaaataaagaaatgttacgttataga<br>tattcatcaaacttaagaacgatttatttactatagtttttttttttttttgctattcaa<br>aattggctaaaaattgtgccaattaaaagctagtaaccgttcaattaataattcttttac<br>aacttgcaaacttttaaactcagcattcttatttcttccctattaaaatttaaaacccat<br>ctatgttctcattaaaactca

R3a	AGCCAAC TGGTGAG ATTAGT<br><br>Start: 240<br>Stop: 260	ATCCTGT ACCCACA ATTTGC<br><br>Start: 382<br>Stop: 363	PGSC0003DMG4000 09455	56	ttctgaaatctgcaaagcaaaacacaaaatgattttaagaaatttgagaagatgaatgtt<br>tctatcttcaattactaacaactttactttacctgttgaatgctaagagtaatttgcaaa<br>caagaatgatcactgatttctctctctcttttgctagtaaatttgcaaacaacaagttgt<br>aactatgtttgctagtgcctattcattgctttctcaattgagtaattgagggtagttggg<br>ggacgttgtacaaaatggggaccatttagacaccaaagtcttgtatcatttaaacatcaa<br>tacaaggttcaattgtgctattttgacatttttggctaattcagccaaatacgttaaagt<br>gtgcaacacactcgcggataacatctcaatatttgaattgcacaaaatgacaaaattact<br>tccatatttgaattgcacaaattactttcctggggtgtttgacaattaatgagtcctgtt<br>ttaaggcttgtagtccttatt
WRKY1	TTTCTTC AGCATCA TCGTC<br><br>Start: 122<br>Stop: 141	TTCCATG CTTGCCA TTAGTC<br><br>Start: 236<br>Stop: 218	PGSC0003DMT4000 56725	57	tttttttgagagatgaaacggaaagaggtatataaaaggttgagatgaagactaaaaaaa<br>aaaagtgtggtcaaagagaagattttgataaaataataacgtaaggtggtgcgtcaaggg<br>tgggcaagtgggaccaagtataaaaaattgtcaagactgagattgacttgttcgcattaa<br>agtaaagagaaagagaaagaagaagaagatgaaacccaccggcccgactagtaaggcgtg<br>atgaaaatgaaaatgaaaaggtagctagtagtgacaacaaagtcaactagttttaagcac<br>aaatttctcgcctcacttacgacttggttattagctaattcatccacccgtttcaattta<br>tataaattagtttaaattcatataaaattttagaaggaaataatgatttttcaaatttat<br>agtctgaaataaatatcccaaaaaatgtaataaataaataaaaatattaccaccccaccc<br>ctaccatcnnnnnnnnnnnnn

Supplementary Table S3. Structure of arginine methyltransferase 5 (PRMT5) inhibitor and the effect of potato leaf treatment with GSK3326595

Name	Chemical Structure	No symptoms of cytotoxicity<br>(72 h after the treatment)
GSK3326595<br>(EPZ015938)<br><br>CAS No.<br>1616392-22-3	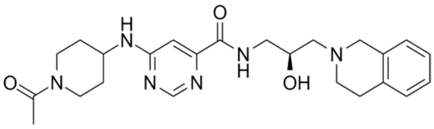	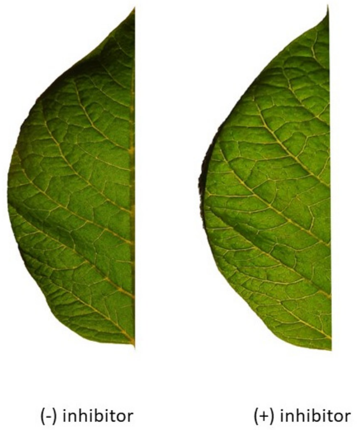
